# The C-Reactive Protein–Albumin–Lymphocyte (CALLY) Index as an Independent Predictor of Progression and Survival in Metastatic Renal Cell Carcinoma

**DOI:** 10.3390/jcm15041475

**Published:** 2026-02-13

**Authors:** Beril Balci Topuz, Ahmet Tufekci, Sibel Cangi, Omer Aydin Yildirim, Mustafa Yildirim

**Affiliations:** 1 Department of Radiation Oncology, Faculty of Medicine, SANKO University, 27090 Gaziantep, Türkiye; 2 Department of Urology, Faculty of Medicine, SANKO University, 27090 Gaziantep, Türkiye; 3 Department of Pathology, Faculty of Medicine, SANKO University, 27090 Gaziantep, Türkiye; 4 Department of Internal Medicine, Faculty of Medicine, SANKO University, 27090 Gaziantep, Türkiye; 5 Department of Medical Oncology, Faculty of Medicine, SANKO University, 27090 Gaziantep, Türkiye

**Keywords:** renal cell carcinoma, index, biomarkers, inflammation

## Abstract

**Background/Objectives**: Systemic inflammation, nutritional status, and immune competence have emerged as important prognostic determinants in metastatic renal cell carcinoma (mRCC). The C-reactive protein–albumin–lymphocyte (CALLY) index integrates these parameters into a single composite biomarker, yet its utility in mRCC remains insufficiently explored. This study aimed to evaluate the prognostic significance of the CALLY index in mRCC and its associations with clinicopathological features and survival outcomes. **Methods**: A total of 68 patients with mRCC treated between 2017 and 2024 were retrospectively analyzed. All patients received first-line VEGF–TKI therapy, and 73.5% subsequently received second-line treatment, predominantly nivolumab. The CALLY index was calculated as (albumin × lymphocyte count)/(CRP × 10^4^), and patients were stratified using the median cut-off (0.16). Survival outcomes were assessed with Kaplan–Meier and Cox regression analyses. Discriminative performance was evaluated using Harrell’s C-index and time-dependent ROC curves at 6, 12, and 24 months. **Results**: Low CALLY (≤0.16) was significantly associated with shorter PFS (4 vs. 8 months, *p* < 0.001) and OS (9 vs. 26 months, *p* < 0.001). In multivariate analysis, the CALLY index independently predicted both PFS (HR = 1.91, *p* = 0.045) and OS (HR = 2.89, *p* = 0.005). Time-dependent ROC analysis demonstrated increasing discriminative strength for PFS (AUC: 0.70 → 0.95) and modest decline for OS over time (AUC: 0.83 → 0.72). CALLY also showed strong associations with IMDC risk classification and peritoneal metastasis. **Conclusions**: The CALLY index is a simple, cost-effective, and objective prognostic biomarker that may independently predict progression and survival in mRCC. Its complementary value to the IMDC model supports its integration into routine risk stratification and real-world clinical decision-making.

## 1. Introduction

Renal cell carcinoma (RCC) accounts for approximately 2–3% of all adult malignancies worldwide and remains one of the leading causes of mortality among genitourinary cancers. The global incidence of RCC continues to rise, primarily due to the increased use of imaging modalities, although mortality rates have plateaued in many high-income countries [1]. At diagnosis, about 70% of patients are present with stage I disease. However, metastatic spread is observed in approximately 10% of newly diagnosed cases, and an additional ~10% of patients initially treated for localized disease eventually develop distant metastases [2,3,4]. Despite advances in targeted therapies and immune checkpoint inhibitors, clinical outcomes in metastatic RCC remain suboptimal, highlighting the urgent need for reliable prognostic biomarkers that can aid in risk stratification and guide treatment decisions.

Beyond tumor-related characteristics [5], growing evidence indicates that host-related factors—particularly systemic inflammation, nutritional status, and immune competence—play a central role in cancer proliferation, angiogenesis, metastatic potential, and immune evasion through sustained interactions between tumor cells and the microenvironment [6]. In clinical practice, this biological interplay is increasingly captured by routinely available serum-based indeces, such as neutrophil-to-lymphocyte ratio (NLR), platelet-to-lymphocyte ratio (PLR), systemic inflammation response index (SIRI), and albumin-to-alkaline phosphatase ratio (AAPR), which have demonstrated prognostic relevance across urogenital malignancies [7,8,9,10]. However, most of these markers reflect a single biological axis and may inadequately represent the combined inflammatory, immune, and nutritional burden that characterizes aggressive disease phenotypes. In this context, easily measurable parameters such as C-reactive protein, serum albumin, and lymphocyte count have emerged as complementary indicators of host–tumor interaction [11,12,13].

The CRP–albumin–lymphocyte (CALLY) index, which integrates CRP, albumin, and lymphocyte count into a single composite score, was initially introduced in studies on hepatocellular carcinoma [14]. Since then, it has demonstrated prognostic value across a wide range of malignancies, including gastrointestinal, lung, and breast cancers [15,16,17,18]. However, evidence supporting its clinical utility in metastatic RCC remains limited. Currently, the most widely used prognostic model is the International Metastatic RCC Database Consortium (IMDC) model, which combines clinical and laboratory variables—such as performance status, hemoglobin, neutrophil and platelet counts, serum calcium, and the interval from diagnosis to systemic therapy initiation—to provide robust prognostic stratification [19]. Nevertheless, incorporating simple, routinely available biomarkers could further enhance predictive accuracy, particularly in heterogeneous patient populations.

Against this background, the objective of our study was to evaluate the prognostic significance of the CALLY index in patients with metastatic RCC, exploring its association with survival outcomes and various clinicopathological features. By comparing CALLY with established prognostic factors, we aimed to assess its potential as an inexpensive, reproducible, and clinically practical tool for routine use in the management of metastatic RCC.

## 2. Materials and Methods

### 2.1. Study Design and Population

This retrospective study included archival metastatic renal cell carcinoma (mRCC) patients diagnosed between 1 January 2017 and 1 January 2024 in the Medical Oncology Department of SANKO University Faculty of Medicine. Ethical approval was obtained from the Scientific Research Evaluation and Ethics Committee of SANKO University Faculty of Medicine (Approval No: 2025/12 and date: 12 November 2025). The study was conducted in accordance with the principles of the Declaration of Helsinki.

All patients received sunitinib or pazopanib as first-line chemotherapy. Patients who progressed received immune checkpoint inhibitor or VEGF–TKI monotherapy as second-line treatment. Treatment decisions were individualized based on each patient’s clinical profile and the availability of therapeutic options. All patients with brain metastases received palliative radiotherapy, while for other metastatic sites, palliative RT was administered only in cases requiring pain relief.

Inclusion criteria comprised adults (≥18 years) with histopathologically verified RCC who either had metastatic disease at presentation or developed metastases during follow-up after an initial diagnosis of non-metastatic RCC. Additional requirements were non-pregnant/non-lactating status, uninterrupted clinical follow-up, and adequate information to compute the CALLY index and determine disease status. Exclusion criteria were cases with active infections, missing clinical/laboratory data, and loss to follow-up within the study period.

We retrospectively reviewed the medical records, clinical assessments, and laboratory data of 86 patients; among them, 68 met the eligibility criteria and were included in the final analysis.

### 2.2. Data Collection

Demographic, clinical, radiological, laboratory, and pathological parameters were retrieved from the hospital’s electronic and archival medical records. Hematologic and biochemical values obtained prior to the initiation of treatment were used to calculate the CALLY index, and their association with disease-related mortality was analyzed. The status of metastatic sites at the time of diagnosis was taken into consideration. Patients were followed up with PET-CT every three months, and disease progression was evaluated according to Response Evaluation Criteria in Solid Tumors (RECIST) 1.1 criteria [20].

CALLY was calculated as (albumin [g/dL] × absolute lymphocyte count [/µL]) ÷ (CRP [mg/dL] × 10^4^). For internal consistency, lymphocyte counts were converted to /µL (multiplying × 10^3^/µL values by 1000) and CRP to mg/dL (dividing mg/L by 10 when needed). Higher CALLY values reflect better nutritional status, lower systemic inflammation, and preserved lymphocyte-mediated immunity.

### 2.3. Statistical Analysis

General statistical analyses were performed using SPSS version 27.0 (IBM Corp.). Advanced survival analyses, including time-dependent ROC curves and Harrell’s concordance index (C-index), were conducted in Python 3.x (Python Software Foundation). Continuous variables were summarized as means or medians with ranges, and categorical variables as frequencies and percentages. Group comparisons were made using the Chi-square or Fisher’s exact tests for categorical variables, and Student’s *t*-test or Mann–Whitney U test for continuous variables, as appropriate.

Overall survival (OS) was defined as the interval from metastatic diagnosis to death or last follow-up, and progression-free survival (PFS) as the time to first documented progression or death. Survival distributions were estimated using the Kaplan–Meier method and compared with the log-rank test.

The CALLY index was evaluated as a dichotomized variable. Candidate cut-off values were initially screened using maximally selected rank statistics; however, the resulting threshold produced markedly unbalanced groups, with very small patient numbers in one stratum. As this may inflate type-I error, reduce statistical power and lead to unstable survival estimates in retrospective cohorts, we adopted the cohort median CALLY value as the final cut-off.

The prognostic impact of clinical, pathological, and laboratory variables was assessed using univariate Cox regression, and variables with *p* < 0.10 were entered into multivariate models. Prior to multivariable modeling, multicollinearity among covariates (including CALLY components, ECOG status, IMDC factors, and metastatic sites) was examined using variance inflation factors (VIFs), with none exceeding the predefined threshold of 3.0.

Model discrimination was evaluated using the C-index. Time-dependent receiver operating characteristic (ROC) analyses were performed to assess the predictive ability of the CALLY index at 6, 12, and 24 months. Two-sided *p*-values < 0.05 were considered statistically significant.

## 3. Results

A total of 68 patients with metastatic RCC were included in the study. The mean age was 61 years, with a median age of 63 years (range: 29–80). For the analyses, patients were stratified by age using 63 years as the cut-off value. The mean follow-up period was 24 months. The median OS was 18 months (95% CI: 13.6–22.4; range: 2–120), while the median PFS was 5 months (95% CI: 4.1–5.9; range: 2–48).

Of the 68 patients, 20 (29.4%) received pazopanib and 48 (70.6%) received sunitinib as first-line therapy. Second-line treatment was administered to 53 patients (77.9%), of whom 40 (75.4%) received nivolumab.

Among 68 patients, 53 (77.9%) underwent nephrectomy, 20 had previously undergone nephrectomy, while the remaining 33 underwent cytoreductive surgery. Lymphovascular invasion (LVI) was identified in 15 patients (22.4%), absent in 52 (77.6%), and undetermined in one case due to missing data. By grade, 5 (7.4%) were grade 1, 22 (32.4%) grade 2, 32 (47.1%) grade 3, and 9 (13.2%) grade 4. Histologically, 53 (77.9%) had clear cell RCC, four of the clear cell cases harbored sarcomatoid features, and 15 (22.1%) had non–clear cell tumors; The median tumor size was 10 cm (range, 5–20 cm).

Only two patients had oligometastatic disease, each with a single metastatic focus in bone and lung, respectively. Metastatic spread most commonly involved distant lymph nodes (75.0%) and the lungs (70.6%), followed by bone (36.8%) and liver (27.9%). Peritoneal implants occurred in 16.2%, and brain metastases were uncommon (5.9%).

### 3.1. Survival Outcomes According to the CALLY Index

**Figure 1:**
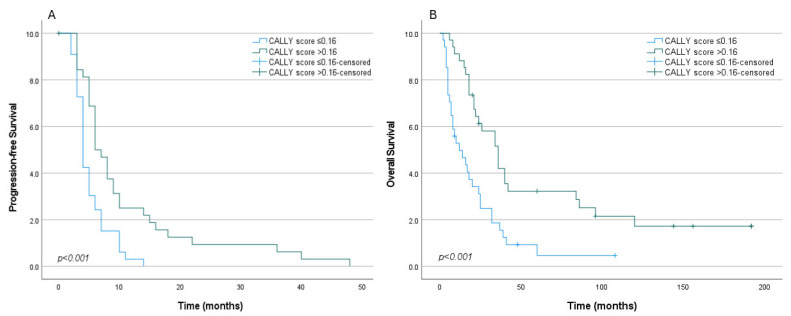
Kaplan–Meier curves for progression-free survival (**A**) and overall survival (**B**) according to the CALLY index (≤0.16 vs. >0.16) in patients with metastatic renal cell carcinoma.

Disease progression was observed in 65 patients. In the low CALLY group [≤0.16 (*n* = 34)], the median PFS was 4 months (95% CI: 3.2–4.8), compared with 8 months (95% CI: 4.3–7.7) in the high CALLY group [>0.16 (*n* = 34)] (*p* < 0.001) (Figure 1A). At 6 months, the PFS rate was 24% in the low CALLY group versus 50% in the high CALLY group. At 12 months, the PFS rate declined to 0% in the low CALLY group, whereas it remained 22% in the high CALLY group. These findings indicate that a low CALLY score is strongly associated with early disease progression and shorter PFS in metastatic RCC.

Patients in the low CALLY group (≤0.16) had a median OS of 9 months (95% CI: 6.3–11.7), whereas those in the high CALLY group (>0.16) had a median OS of 26 months (95% CI: 10.8–41.2). This difference was statistically significant (log-rank test, *p* < 0.001) (Figure 1B). The 12-month OS rate was 36.7% in the low CALLY group compared with 88.2% in the high CALLY group. At 24 months, survival rates were 22.9% vs. 52.4% at 36 months 3.0% vs. 31.4%, and at 60 months 4.7% vs. 19.6%, respectively. These findings demonstrate that a low CALLY score is strongly associated with early mortality.

**Figure 2:**
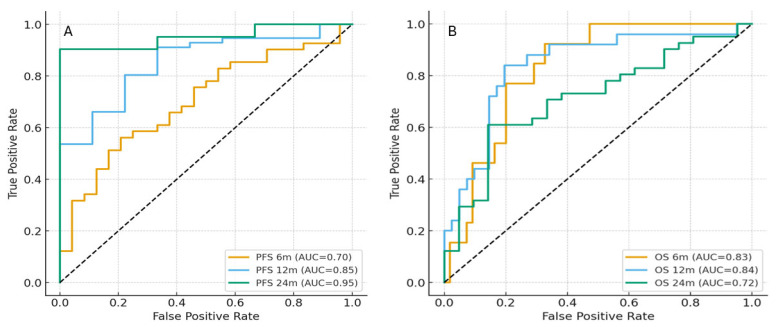
Time-dependent receiver operating characteristic (ROC) curves of the CALLY index for predicting progression-free survival (**A**) and overall survival (**B**) at 6, 12, and 24 months.

Time-dependent ROC analysis showed that the CALLY index retained good discrimination across follow-up; the corresponding AUCs were 0.70, 0.85, and 0.95 for PFS, respectively (Figure 2A). For OS, AUCs were 0.83 at 6 months, 0.84 at 12 months, and 0.72 at 24 months (Figure 2B). The C-index was 0.73 for OS and 0.66 for PFS, indicating fair-to-good discriminative ability of the CALLY index.

### 3.2. Survival Outcomes According to the IMDC Risk Classification

Based on the IMDC risk stratification, 4 patients (5.9%) were categorized as favorable risk, 39 (57.4%) as intermediate risk, and 25 (36.8%) as poor risk. For statistical analyses, the favorable group was combined with the intermediate risk group. A clear separation in survival outcomes was observed. In the poor-risk group, the median OS was 7 months (95% CI: 4.6–9.4), with Kaplan–Meier estimates showing survival rates of 56% at 6 months and 14% at 12 months. In contrast, the favorable/intermediate-risk group had a median OS of 26 months (95% CI: 19.2–32.8), with survival rates of 90.6%, 88.2%, 58.5%, 27.9%, and 15.6% at 6, 12, 24, 36, and 60 months, respectively.

### 3.3. Associations Between CALLY Index and Clinicopathological Variables

No significant association was observed between CALLY groups and age (χ^2^ = 0.94, *p* = 0.33), sex (χ^2^ = 1.439, *p* = 0.23), or ECOG performance status (χ^2^ = 3.46, *p* = 0.18). In contrast, lymphovascular invasion (LVI) was significantly more frequent in the low CALLY group (χ^2^ = 3.945, *p* = 0.047). A strong association was also noted with the IMDC risk classification (χ^2^ = 14.233, *p* < 0.001), as patients with low CALLY scores were predominantly in the poor-risk group, whereas those with high CALLY scores were more often in the favorable/intermediate-risk group. Histological subtype (clear cell vs. non-clear cell; χ^2^ = 0.770, *p* = 0.380) was not associated with CALLY, while a borderline trend was observed for Fuhrman grade, with high-grade tumors being more common in the low CALLY group (χ^2^ = 3.010, *p* = 0.083). The association between nephrectomy status and CALLY groups was not statistically significant (Pearson’s Chi-square = 0.770, *p* = 0.380). (Table 1).

Regarding metastatic sites, lung metastases were significantly more common in the low CALLY group (χ^2^ = 7.083, *p* = 0.008), while a trend toward higher bone metastasis was also noted (χ^2^ = 3.100, *p* = 0.078). No associations were found for liver metastasis (χ^2^ = 1.826, *p* = 0.177), brain metastasis (χ^2^ = 1.063, *p* = 0.303), or lymph node metastasis (χ^2^ = 0.078, *p* = 0.779). However, when examining the distribution of the cases, 12 of the 19 patients with liver metastases and 16 of the 25 patients with bone metastases were numerically more frequent in the low CALLY group. Finally, peritoneal metastasis was significantly associated with low CALLY scores (χ^2^ = 5.314, *p* = 0.021) (Table 1).

### 3.4. Univariable and Multivariable Cox Regression Analyses for PFS and OS

For PFS, univariate analysis demonstrated that low CALLY index (≤0.16 vs. >0.16), poor ECOG performance status (2–3 vs. 0–1), IMDC poor risk group (poor vs. favorable/intermediate), non-clear cell histology (non-clear vs. clear), presence of LVI (yes vs. no), absence of surgery (no vs. yes), and lymph node metastasis (yes vs. no) were all associated with an increased risk of progression. In the multivariate model, the CALLY index (HR = 1.91, *p* = 0.045) and lymph node metastasis (HR = 2.94, *p* = 0.002) remained independent predictors of progression (Table 2).

For OS, univariate analysis identified low CALLY index (≤0.16 vs. >0.16), poor ECOG performance status (2–3 vs. 0–1), IMDC poor risk (poor vs. favorable/intermediate), high Fuhrman grade (3–4 vs. 1–2), presence of LVI (yes vs. no), absence of surgery (no vs. yes), and the presence of lung, bone, and peritoneal metastases (yes vs. no) as adverse prognostic factors. In the multivariate analysis, CALLY index (HR = 2.89, *p* = 0.005), poor ECOG performance status (HR = 8.0, *p* = 0.022), IMDC poor risk (HR = 5.2, *p* < 0.001), lung metastasis (HR = 2.38, *p* = 0.034), bone metastasis (HR = 2.17, *p* = 0.046), and lymph node metastasis (HR = 3.70, *p* < 0.001) were retained as independent predictors of poor survival (Table 3).

## 4. Discussion

Our study demonstrates that the CALLY index serves as a significant prognostic biomarker in patients with mRCC, showing robust predictive value for both PFS and OS. These results emphasize the clinical relevance of systemic inflammatory and nutritional indices in risk stratification of mRCC, supporting the growing evidence that inflammation-immunity balance has a decisive influence on cancer progression and treatment outcomes.

The biological relevance of CALLY may be particularly pronounced in RCC, a tumor characterized by close interactions between angiogenesis, immune modulation, and systemic inflammation. Elevated pro-inflammatory cytokines, including IL-6, have been associated with poorer survival in RCC, underscoring the prognostic importance of immune modulation in this tumor type [21]. Dysregulation of the VHL–HIF pathway promotes pro-inflammatory and pro-angiogenic signaling, contributing to elevated CRP levels and increased systemic inflammatory burden [22]. In parallel, the immunogenic nature of RCC renders lymphocyte-mediated antitumor immunity a critical determinant of clinical outcome, while hypoalbuminemia reflects cancer-related catabolism and metabolic vulnerability frequently observed in advanced diseases [10]. By integrating these RCC-specific inflammatory, immune, and metabolic dimensions, the CALLY index may capture tumor–host interactions that are closely associated with disease aggressiveness in mRCC.

The variation in optimal CALLY cut-off values reported across studies likely reflects tumor-specific biology and disease stage differences. For example, Hirata et al. [23] reported a threshold of 1.28 based on the cohort median in surgically resected RCC, where metastasis was uncommon, whereas a lower value (0.12) using maximally selected rank statistics was proposed in a Turkish mRCC cohort [24]. Our median-based cut-off (0.16) lies within this range, supporting its applicability in advanced disease. Importantly, the challenge of defining standardized biomarker cut-offs should also be interpreted within the broader context of metastatic RCC, where several aspects of clinical management—such as the role and timing of cytoreductive nephrectomy—remain subjects of ongoing debate. As highlighted by Napolitano et al., the heterogeneity of disease biology, treatment strategies, and patient selection complicates the establishment of universal thresholds even for well-established clinical decisions [25]. In this evolving landscape, inflammation- and nutrition-based biomarkers such as CALLY are likely to require disease- and stage-specific calibration rather than a single universally applicable cut-off.

Under VEGF-TKI–based therapy, reported median OS values were approximately 43 months in the IMDC favorable-risk, 23 months in intermediate-risk, and 8 months in poor-risk groups [19]. With the rapid evolution of immunotherapy, dual ICI regimens have become standard first-line options in updated international guidelines, regardless of risk status. Nevertheless, according to the CheckMate 214 trial, the ipilimumab + nivolumab combination did not confer an OS benefit over sunitinib in favorable-risk patients [26]. Recent IMDC data confirm that these risk groups retain prognostic value in the immunotherapy era, underscoring the need for biomarker-based approaches to optimize first-line treatment selection [27,28,29,30].

Despite these advances, access to immunotherapy remains limited in many healthcare systems, primarily due to reimbursement constraints. In Türkiye, national reimbursement regulations still restrict the use of immunotherapy in clinical practice [31]; since July 2025, the combination of nivolumab and ipilimumab has been reimbursed, but ipilimumab is covered for only four cycles. Consequently, all patients in our cohort received VEGF–TKI therapy as first-line treatment, providing a valuable real-world context for evaluating alternative biomarkers such as CALLY.

Consistent with prior literature [23,24], the CALLY index in our study correlated strongly with IMDC risk categories. Median OS was 26 months in the combined favorable/intermediate-risk cohort and 7 months in the poor-risk group, mirroring previous IMDC-based survival gradients. Similarly, patients with high CALLY values had a median OS of 26 months versus 9 months in the low CALLY group, suggesting that CALLY reflects similar biological aggressiveness through a simpler, cost-effective laboratory measure.

Taken together, integrating CALLY with the IMDC framework may provide additional biological context to conventional risk stratification in mRCC. Given the marked heterogeneity of the IMDC intermediate-risk group, CALLY may help uncover biological variation within the same risk category, whereby low CALLY scores reflect a more fragile phenotype with a higher propensity for early progression, supporting the “risk-within-risk” concept. In addition, CALLY may help identify a small subset of IMDC favorable-risk patients with a biologically vulnerable profile. Considering that dual immunotherapy has not demonstrated an additional OS benefit in favorable-risk patients in the CheckMate-214 trial, this observation is of particular clinical relevance [26]. In such cases, a low CALLY score—reflecting a catabolic and pro-inflammatory state with reduced immune reserve—may identify patients who could benefit from individualized therapeutic strategies, closer surveillance, and early supportive interventions, thereby positioning CALLY as a complementary marker to IMDC in routine clinical decision-making. However, given the single-center design and relatively small cohort size of the present study, these findings should be interpreted with caution, and the potential clinical integration of CALLY requires validation in larger, multicenter populations.

The discriminative ability of the CALLY index for PFS appeared to increase over time, with AUC values rising from 0.70 at 6 months to 0.95 at 24 months. This pattern suggests that the prognostic separation between low- and high-CALLY groups becomes more evident during longer follow-up, possibly reflecting earlier progression in patients with low CALLY scores and more sustained disease control among those with higher scores. In contrast, the discriminative ability of CALLY for OS tended to decrease over time (AUC 0.83 at 6 months vs. 0.72 at 24 months), suggesting that the index may be more informative for early mortality risk, whereas long-term survival is likely influenced by additional factors such as subsequent treatment lines and competing comorbidities.

Regarding metastatic distribution, our cohort’s profile was broadly consistent with previous reports [32,33], though the proportion of distant lymph node involvement was relatively high. Previous nomogram-based studies in patients with primary metastatic renal cell carcinoma have consistently demonstrated that liver and brain metastases confer the poorest survival outcomes, and lung and bone metastases are also associated with unfavorable overall survival. Although brain and liver metastases were associated with overall survival in univariate analyses in our analysis, this association did not persist as an independent prognostic factor after adjustment for confounding variables in the multivariate model. In contrast, bone and lung metastases remained significant independent prognostic factors for OS, in line with findings from previous data [34]. The relatively low number of patients with brain and liver metastases compared to bone and lung metastases in our cohort may have limited the statistical power to detect their independent prognostic impact in multivariate models.

Interestingly, CALLY and distant lymph node metastasis independently predicted outcomes but were not correlated. Although lymphatic dissemination interacts with the host immune system through antigen presentation and T-cell activation, these are localized phenomena that may not translate into measurable systemic markers. Because CALLY reflects the global inflammatory and catabolic state—largely driven by visceral and peritoneal tumor burden—this biological distinction may explain why both parameters independently predict survival while showing no significant statistical correlation. The observed association between low CALLY and peritoneal metastasis further supports this concept, as peritoneal spread reflects both high systemic inflammation and aggressive tumor biology.

This study has several limitations. Its retrospective design and single-center nature may introduce selection bias. The sample size, particularly in subgroup analyses such as peritoneal and brain metastases, was limited, which may have reduced the statistical power to detect certain associations. Moreover, no external validation cohort was available to confirm the generalizability of the proposed cut-off values. Laboratory measurements were obtained at baseline only; thus, dynamic changes in CALLY over the treatment course could not be evaluated. Although all patients in our cohort received VEGF–TKI-based first-line therapy, reflecting real-world practice under local reimbursement constraints, this treatment uniformity may be considered both a strength and a limitation of the study. While it enhances the relevance of our findings in healthcare settings where access to immunotherapy remains restricted, it may limit the generalizability of our results to regions where immune checkpoint inhibitor–based combinations constitute the standard first-line approach. Future prospective and multicenter studies with larger cohorts are needed to validate our findings and explore longitudinal CALLY variations.

## 5. Conclusions

This study demonstrates that the CALLY index is an independent prognostic biomarker for both progression-free and overall survival in patients with metastatic renal cell carcinoma. Lower CALLY values were associated with more aggressive disease features, higher metastatic burden, and poorer survival, showing close concordance with adverse IMDC risk classification. Time-dependent analyses further suggest that CALLY has clinically meaningful discriminative ability, particularly for early disease progression and early mortality.

Given its simplicity, objectivity, and reliance on routinely available laboratory parameters, CALLY may represent a practical adjunct to established prognostic models in real-world clinical settings. However, considering the retrospective, single-center design and limited sample size of the present study, these findings should be interpreted cautiously. Prospective, multicenter investigations are warranted to validate the prognostic utility of CALLY and to determine whether dynamic changes in the index during systemic therapy could further refine risk stratification and clinical decision-making in mRCC.

## Figures and Tables

**Table 1:** Baseline demographic, clinical, pathological, and metastatic characteristics of patients stratified by CALLY index (≤0.16 vs. >0.16).

Characteristic	Category	CALLY ≤ 0.16 *n* (%)	CALLY > 0.16 *n* (%)	*p*-Value
Age	≤63	19 (55.9)	15 (44.1)	0.330
	>63	15 (44.1)	19 (55.9)	
Gender	Female	5 (14.7)	9 (26.5)	0.230
	Male	29 (85.3)	25 (73.5)	
ECOG	0–1	28 (82.3)	34 (100)	0.180
	2–3	6 (17.7)	0 (0)	
IMDC risk	Favorable/Intermediate	**14 (41.2)**	**29 (85.3)**	**<0.001**
	Poor	**20 (58.8)**	**5 (14.7)**	
Histology	Clear	28 (82.4)	25 (73.5)	0.380
	Non-clear	6 (17.6)	9 (26.5)	
Fuhrman grade	1–2	10 (29.4)	17 (50)	0.083
	3–4	24 (70.6)	17 (50)	
LVI	No	**23 (67.6)**	**29 (87.9)**	**0.047**
	Yes	**11 (32.4)**	**4 (12.1)**	
Nephrectomy	No	9 (26.5)	6 (17.6)	0.380
	Yes	25 (73.5)	28 (82.4)	
Liver metastasis	No	22 (64.7)	27 (79.4)	0.177
	Yes	12 (35.3)	7 (20.6)	
Lung metastasis	No	**5 (14.7)**	**15 (44.1)**	**0.008**
	Yes	**29 (85.3)**	**19 (55.9)**	
Bone metastasis	No	18 (52.9)	25 (73.5)	0.078
	Yes	16 (47.1)	9 (26.5)	
Brain metastasis	No	31 (91.2)	33 (97.1)	0.303
	Yes	3 (8.8)	1 (2.9)	
Peritoneal metastasis	No	**25 (73.5)**	**32 (94.1)**	**0.021**
	Yes	**9 (26.5)**	**2 (5.9)**	
Distant lymph node metastasis	No	8 (23.5)	9 (26.5)	0.779
	Yes	26 (76.5)	25 (73.5)	

Abbreviations: CALLY (CRP–albumin–lymphocyte index); ECOG (Eastern Cooperative Oncology Group performance status); IMDC (International Metastatic RCC Database Consortium risk group); LVI (Lymphovascular invasion). Bold values indicate statistically significant results.

**Table 2:** Univariate and multivariate Cox regression analyses for progression-free survival (PFS) according to clinical, pathological, and metastatic variables, including the CALLY index.

	Univariate HR (95% CI)	*p* Value	Multivariate HR (95% CI)	*p* Value
**CALLY (≤0.16 vs. >0.16)**	2.22 (1.30–3.79)	**0.003**	1.91 (0.98–3.33)	**0.045**
**Age (>63 vs. ≤63)**	1.31 (0.79–2.19)	0.297	-	-
**Gender (Male vs. Female)**	0.74 (0.41–1.35)	0.331	-	-
**ECOG (2–3 vs. 0–1)**	1.87 (1.19–2.94)	**0.006**	0.79 (0.80–2.91)	0.736
**IMDC risk (poor vs. favorable/intermediate)**	2.71 (1.53–4.80)	**<0.001**	1.51 (0.79–2.89)	0.209
**Histology (non-clear vs. clear)**	2.05 (1.10–3.82)	**0.024**	1.29 (0.66–2.52)	0.454
**Fuhrman grade (3–4 vs. 1–2)**	0.69 (0.41–1.16)	0.164	-	-
**LVI (yes vs. no)**	2.33 (1.25–4.36)	**0.008**	-	-
**Nephrectomy (no vs. yes)**	2.26 (1.23–4.16)	**0.008**	1.35 (0.59–3.03)	0.475
**Liver metastasis (yes vs. no)**	1.57 (0.89–2.76)	0.121	-	-
**Lung metastasis (yes vs. no)**	1.24 (0.73–2.13)	0.425	-	-
**Bone metastasis (yes vs. no)**	1.41 (0.84–2.38)	0.196	-	-
**Brain metastasis (yes vs. no)**	1.36 (0.49–3.78)	0.551	-	-
**Peritoneal metastasis (yes vs. no)**	1.72 (0.88–3.36)	0.112	-	-
**Distant Lymph node metastasis (yes vs. no)**	2.33 (1.24–4.37)	**0.008**	2.94 (1.47–5.88)	**0.002**

Abbreviations: CALLY (CRP–albumin–lymphocyte index); ECOG (Eastern Cooperative Oncology Group performance status); IMDC (International Metastatic RCC Database Consortium risk group); LVI (Lymphovascular invasion). Multivariable Cox models included key clinical covariates and variables with *p* < 0.10 in univariable analysis. *p*-value below 0.05 were considered statistically significant. Bold values indicate statistically significant results.

**Table 3:** Univariate and multivariate Cox regression analyses for overall survival (OS) according to clinical, pathological, and metastatic variables, including the CALLY index.

	Univariate HR (95% CI)	*p* Value	Multivariate HR (95% CI)	*p* Value
**CALLY (≤0.16 vs. >0.16)**	3.51 (2.00–6.16)	**<0.001**	2.89 (1.36–6.15)	**0.005**
**Age (>63 vs. ≤63)**	1.69 (0.99–2.88)	**0.057**	1.72 (0.92–3.22)	0.087
**Gender (Male vs. Female)**	0.92 (0.48–1.75)	0.800	-	-
**ECOG (2–3 vs. 0–1)**	2.12 (1.15–3.90)	**0.005**	8.0 (1.35–45.4)	**0.022**
**IMDC risk (poor vs. favorable/intermediate)**	10.1 (7.90–21.20)	**<0.001**	5.2 (3.0–15.30)	**<0.001**
**Histology (non-clear vs. clear)**	1.61 (0.85–3.06)	0.148	-	-
**Fuhrman grade (3–4 vs. 1–2)**	1.83 (1.05–3.20)	**0.033**	1.45 (0.71–2.99)	0.311
**LVI (yes vs. no)**	2.52 (1.36–4.69)	**0.003**	1.83 (0.84–3.98)	0.130
**Nephrectomy (no vs. yes)**	2.68 (1.376–5.242)	**0.004**	1.55 (0.55–4.31)	0.400
**Liver metastasis (yes vs. no)**	1.77 (0.996–3.13)	**0.052**	1.17 (0.58–2.33)	0.662
**Lung metastasis (yes vs. no)**	2.16 (1.18–3.94)	**0.012**	2.38 (1.02–5.26)	**0.034**
**Bone metastasis (yes vs. no)**	2.85 (1.65–4.91)	**<0.001**	2.17 (1.01–4.55)	**0.046**
**Brain metastasis (yes vs. no)**	2.64 (0.93–7.48)	**0.069**	1.38 (0.43–4.43)	0.590
**Peritoneal metastasis (yes vs. no)**	3.92 (1.93–7.98)	**<0.001**	1.14 (0.45–2.88)	0.779
**Distant lymph node metastasis (yes vs. no)**	0.57 (0.31–1.07)	**0.080**	3.70 (1.52–8.93)	**<0.001**

Abbreviations: CALLY (CRP–albumin–lymphocyte index); ECOG (Eastern Cooperative Oncology Group performance status); IMDC (International Metastatic RCC Database Consortium risk group); LVI (Lymphovascular invasion). Multivariable Cox models included key clinical covariates and variables with *p* < 0.10 in univariable analysis. *p*-values below 0.05 were considered statistically significant. Bold values indicate statistically significant results.

## Data Availability

The raw data supporting the conclusions of this article will be made available by the authors on request.

## Abbreviations

AAPR: Albumin-to-Alkaline Phosphatase Ratio; AUC: Area Under the Curve; CALLY: C-Reactive Protein–Albumin–Lymphocyte Index; CI: Confidence Interval; C-index: Concordance Index; CRP: C-Reactive Protein; ECOG: Eastern Cooperative Oncology Group Performance Status; HIF: Hypoxia-Inducible Factor; HR: Hazard Ratio; ICI: Immune Checkpoint Inhibitor; IMDC: International Metastatic Renal Cell Carcinoma Database Consortium; IL-6: Interleukin-6; LVI: Lymphovascular Invasion; mRCC: Metastatic Renal Cell Carcinoma; NLR: Neutrophil-to-Lymphocyte Ratio; OS: Overall Survival; PET-CT: Positron Emission Tomography–Computed Tomography; PFS: Progression-Free Survival; PLR: Platelet-to-Lymphocyte Ratio; RCC: Renal Cell Carcinoma; RECIST: Response Evaluation Criteria in Solid Tumors; ROC: Receiver Operating Characteristic; RT: Radiotherapy; SII: Systemic Immune-Inflammation Index; SIRI: Systemic Inflammation Response Index; TKI: Tyrosine Kinase Inhibitor; VEGF: Vascular Endothelial Growth Factor; VHL: Von Hippel–Lindau; VIF: Variance Inflation Factor.
